# Magnitude, Frequency, and Accumulation: Workload Among Injured and Uninjured Youth Basketball Players

**DOI:** 10.3389/fspor.2021.607205

**Published:** 2021-04-06

**Authors:** Lauren C. Benson, Oluwatoyosi B. A. Owoeye, Anu M. Räisänen, Carlyn Stilling, W. Brent Edwards, Carolyn A. Emery

**Affiliations:** ^1^Sport Injury Prevention Research Centre, Faculty of Kinesiology, University of Calgary, Calgary, AB, Canada; ^2^United States Olympic and Paralympic Committee, Colorado Springs, CO, United States; ^3^Department of Physical Therapy and Athletic Training, Doisy College of Health Sciences, Saint Louis University, Saint Louis, MO, United States; ^4^Department of Physical Therapy Education, College of Health Sciences, Western University of Health Sciences, Lebanon, OR, United States; ^5^Faculty of Kinesiology, University of Calgary, Calgary, AB, Canada; ^6^McCaig Institute for Bone and Joint Health, University of Calgary, Calgary, AB, Canada; ^7^Department of Community Health Sciences, Cumming School of Medicine, University of Calgary, Calgary, AB, Canada; ^8^Alberta Children's Hospital Research Institute, University of Calgary, Calgary, AB, Canada; ^9^Department of Pediatrics, Cumming School of Medicine, University of Calgary, Calgary, AB, Canada

**Keywords:** load, jump, overuse, inertial measurement unit, principal components analysis

## Abstract

Overuse injuries are common in basketball. Wearable technology enables the workload to be monitored in sport settings. However, workload–injury models lack a biological basis both in the metrics recorded and how workload is accumulated. We introduce a new metric for monitoring workload: weighted jump height, where each jump height is weighted to represent the expected effect of the jump magnitude on damage to the tendon. The objectives of this study were to use principal components analysis to identify distinct modes of variation in all workload metrics accumulated over 1, 2, 3, and 4 weeks and to examine differences among the modes of variation in workload metrics between participants before the injury and uninjured participants. Forty-nine youth basketball players participated in their typical basketball practices and games, and lower extremity injuries were classified as patellar or Achilles tendinopathy, other overuse, or acute. An inertial measurement unit recorded the number and height of all jumps, and session rating of perceived exertion was recorded. The previous 1-, 2-, 3-, and 4-week workloads of jump count, jump height, weighted jump height, and session rating of perceived exertion were summed for each participant-week. Principal components analysis explained the variance in the accumulated workload variables. Using the retained principal components, the difference between the workload of injured participants in the week before the injury and the mean workload of uninjured participants was described for patellar or Achilles tendinopathy, overuse lower extremity injury, and any lower extremity injury. Participants with patellar or Achilles tendinopathy and overuse lower extremity injuries had a low workload magnitude for all variables in the 1, 2, 3, and 4 weeks before injury compared with the weeks before no injury. Participants with overuse lower extremity injuries and any lower extremity injury had a high previous 1-week workload for all variables along with a low previous 3- and 4-week jump count, jump height, and weighted jump height before injury compared with the weeks before no injury. Weighted jump height represents the cumulative damage experienced by tissues due to repetitive loads. Injured youth basketball athletes had a low previous 3- and 4-week workloads coupled with a high previous 1-week workload.

## Introduction

Basketball is one of the top three team sports for youth participation in North America (Emery et al., 2006; Solutions Research Group, 2014; NFHS, 2015; Turner et al., 2015). Basketball is a high-intensity sport in which players perform repetitive movements, including frequent jumping and changing of direction (Abdelkrim et al., 2007). As such, knee and ankle injuries, specifically, overuse (i.e., gradual onset) injuries such as patellar and Achilles tendinopathies are common (Cook et al., 2000; Borowski et al., 2008; Zwerver et al., 2011; Simpson et al., 2016; Leppänen et al., 2017; Owoeye et al., 2019). Athletic injury frameworks aim to identify the relationship between repetitive loads placed on tissues of the body and injury (Edwards, 2018; Kalkhoven et al., 2020), which can inform workload modification strategies for the prevention of overuse injuries (Soligard et al., 2016). Wearable technology has made it possible to record a variety of workload metrics in sport settings, and this facilitates the analysis of the workload–injury relationship (Drust et al., 2007; Taylor et al., 2012; Halson, 2014; Benson et al., 2018; Impellizzeri et al., 2019). However, workload–injury models have been criticized for lacking a biological basis both in the metrics recorded and how workload is accumulated over time to represent adaptations in the body due to fitness and fatigue (Impellizzeri et al., 2020a; Kalkhoven et al., 2020; Wang et al., 2020). Furthermore, previous investigations have used multiple similar metrics (e.g., total distance, high-speed distance) and redundant accumulation methods [e.g., acute–chronic workload ratio (ACWR), 2- and 3-week cumulative workloads] to generate many highly correlated workload variables, which may contribute to the inconclusive evidence regarding the relationship between workload and injury (Benson et al., 2020a). For example, several systematic reviews have identified a relationship between the ACWR and injury (Drew and Finch, 2016; Jones et al., 2017; Eckard et al., 2018; Griffin et al., 2020), but these summaries may be subject to selective reporting bias (Impellizzeri et al., 2020c). Additionally, the only study to randomize a training load intervention based on the ACWR found no effect on health problems in athletes (Dalen-Lorentsen et al., 2020). Establishing workload metrics that represent the cumulative damage experienced by tissues in the body subjected to repetitive loads and the appropriate timeframe for positive tissue adaptation may improve the understanding of how workload patterns influence injury.

During sport, the tissues in the body are exposed to stresses and strains (Wang et al., 2020) that provide the stimulus for adaptation (Impellizzeri et al., 2019; Wang et al., 2020). Functional adaptive responses such as tissue remodeling with appropriate recovery lead to improvements in physical performance, injury resistance, and health (Impellizzeri et al., 2019). However, when the stress and strain applied during exercise exceeds the material strength of the tissue, either in a single high-magnitude event or through repeated loading at lower magnitudes, an injury will occur (Edwards, 2018; Kalkhoven et al., 2020). Importantly, mechanical models demonstrate that damage to a tissue, and therefore the risk of injury, is greater with higher magnitude than with a higher frequency of loading (Edwards, 2018).

A framework for understanding athletic injury should consider both the physiological and mechanical characteristics of the body (Kalkhoven et al., 2020). Direct measurement of tissue stress and strain is not practical in a sport setting; thus, the external load is often estimated by measuring the external forces applied during exercise (Impellizzeri et al., 2019). In jumping sports, common measures of external load include the frequency (count) and magnitude (height) of jumps (Moran et al., 2019). Both a high jump count (Visnes and Bahr, 2013) and a high jump height (Visnes et al., 2013) have been associated with the development of overuse knee injuries. However, in these studies, the frequency of jumps was estimated by training volume, and the jump magnitude was only recorded during baseline testing of maximal jumping ability. Recording jump count and height for all jumps during training and competition and adjusting workload metrics such that higher load magnitudes represent greater tendon damage (Kiernan et al., 2018; Firminger et al., 2020) should improve the ability to identify the association between workload and overuse injuries such as patellar and Achilles tendinopathies. Additionally, objective (e.g., heart rate) or subjective [e.g., rating of perceived exertion (RPE)] measures of internal load represent the psychophysiological response to an external load (Impellizzeri et al., 2019). It is expected that the internal load will be proportional to the external load; therefore, the relationship between external and internal load can be used as an indicator of an athlete's balance of fitness and fatigue (Halson, 2014; Vanrenterghem et al., 2017; Impellizzeri et al., 2019; Ryan et al., 2020).

As time is a factor in how tissue adapts to a training stimulus (Impellizzeri et al., 2019; Wang et al., 2020), the balance of fitness and fatigue is also modeled by exposure to the workload over acute and chronic periods, with acute workload representing fatigue or a negative training effect and chronic workload representing fitness or a positive training effect. This model of performance is often extrapolated to represent injury risk when the ACWR indicates that workload in the acute period is greater or less than the average workload in the chronic period (Soligard et al., 2016). Typically, the acute and chronic periods used are 1 and 4 weeks, respectively, despite no scientific basis for choosing these periods uniformly for all levels of athletes or the assumption that all tissues have the same time course for adaptation (Impellizzeri et al., 2020a). In fact, when the performance model of fitness and fatigue was adapted for injury risk, Hulin et al. (2014) indicated that the choice of 1 and 4 weeks was arbitrarily chosen to align with common periodization in training and may not accurately represent the training–stress balance for biological tissue. Other periods and methods for relating acute and chronic workloads have been explored (Williams et al., 2017b) but still suffer from a lack of scientific basis and mathematical shortcomings (Impellizzeri et al., 2020a,b,c; Wang et al., 2020; Dalen-Lorentsen et al., 2021). In some cases, investigators have used hundreds of similar combinations of accumulated workload variables to investigate the workload–injury relationship (Bowen et al., 2017, 2020).

Rather than choosing *a priori* the workload metrics and methods for representing the accumulation of workload, principal components analysis (PCA) is an unbiased dimensionality reduction tool that can reduce a dataset to its major modes of variation (Jackson, 2003; Brandon et al., 2013). PCA has been used to identify distinct workload information from large datasets (Williams et al., 2017a; Weaving et al., 2018). PCA can rank each data point according to the major modes of variation within the dataset, which can then be used in subsequent analyses in lieu of the original variables.

In this paper, we introduce a new metric for monitoring workload in basketball: weighted jump height, where each jump height is weighted to represent the expected effect of the jump magnitude on damage to the tendon (Edwards, 2018). Weighted jump height was recorded along with unweighted measures of external load, internal load, and all injuries for an entire season of youth basketball. The objectives of this study were to use PCA to identify distinct modes of variation in all workload metrics accumulated over 1, 2, 3, and 4 weeks and to examine differences among the modes of variation in workload metrics between participants before the injury and uninjured participants according to three injury classifications: patellar or Achilles tendinopathy, overuse lower extremity injury, and any lower extremity injury. It was expected that (i) weighted jump height would represent a unique metric relative to unweighted measures of external load and internal load, (ii) PCA would eliminate the redundancy created by accumulating workload over several iterations of multiple weeks, and (iii) participants with patellar or Achilles tendinopathy and other overuse lower extremity injuries would have a greater jump magnitude in the week before injury than uninjured participants.

## Methods

### Participants and Exposure

High school basketball players from four (two females, two males) teams in Calgary, AB, participated in this cohort study. Baseline characteristics included sex, age, mass, height, leg length, and 12-month injury history. Participants participated in their typical basketball practice and game sessions during the season (December 2017-March 2018).

After each game and practice, a team designate gathered information regarding team session duration, player participation levels [full, partial (less than 75% of team session duration), or no participation], and reasons for partial and no participation (injured, sick, or absent). Session duration for players with partial participation was recorded as 50% of the team session duration. Team session duration was defined as active exposure time, including time spent in the warm-up, playing, and cool-down. Unstructured individual or peer-led time on court (e.g., pickup games, physical education class) did not constitute organized basketball for this study (Leppänen et al., 2015).

### Workload Monitoring

Participants were asked to wear a commercially available inertial measurement unit consisting of a tri-axial accelerometer, gyroscope, and magnetometer (VERT®, Mayfonk Inc., Fort Lauderdale, FL, USA) during each practice and game session. Participants secured the device in an elastic waistband and positioned it near the center of mass according to the manufacturer's instructions. As the VERT® recorded movement patterns, the data were transferred in real-time *via* Bluetooth 4.0 technology to an associated Apple iPad application (iPad Air 2, Apple, Cupertino, CA, USA; VTS Basic, Mayfonk Inc., Fort Lauderdale, FL, USA; VERTcoach, version 2.2.6, Mayfonk Inc., Fort Lauderdale, FL, USA), which processed the data using proprietary algorithms and reported the number and height of jumps over six inches (15.24 cm). The use of this device for recording jump count and jump height has been previously validated in youth basketball and youth and elite volleyball (Charlton et al., 2017; MacDonald et al., 2017; Skazalski et al., 2018; Benson et al., 2020b). The output variables were stored on and later accessed from a server (myVERT® BETA, Mayfonk Inc., Fort Lauderdale, FL, USA) maintained by the product manufacturer. Additionally, participants reported their RPE on a Borg CR-10 scale (Foster et al., 1996; Lupo et al., 2017) to the team designate within 10 min after the end of each session (Uchida et al., 2014; Fanchini et al., 2015; Christen et al., 2016; Castagna et al., 2017).

### Injury Surveillance

This study is part of a larger study that evaluated all-complaint injuries (i.e., acute and overuse/gradual onset injuries) in a cohort of youth basketball players; the details of the injury surveillance methods used in the current study have been described in the larger cohort study (Owoeye et al., 2020). An injury was defined as: any physical complaint, including pain, ache, joint instability, stiffness, or any other complaint resulting from participating in basketball-related activities, including but irrespective of the need for medical attention (seeking care from a trainer or medical practitioner) or time loss (inability to complete a basketball-related session or participate in one or more days after the onset of injury) (Fuller et al., 2006; Clarsen et al., 2013; Timpka et al., 2014; Harøy et al., 2017; Owoeye et al., 2018, 2020). Injuries were identified as acute if their onset was sudden and clearly associated with an identifiable event and overuse if their onset was gradual and was unidentifiable with a particular event (Fuller et al., 2007; Bahr et al., 2020). In addition, recurrent injuries were defined as repeat episodes of index injuries previously reported by participants during the study period (Fuller et al., 2007; Bahr et al., 2020).

Injury data were collected across a combination of three pragmatic methods involving a daily injury registration by team designates, weekly self-report of knee and ankle injuries by players, and a follow-up phone interview by the study physical therapist or trained research assistant when no injury information was available (Owoeye et al., 2020). In the current study, and as part of the all-complaint injury methodology used in the larger cohort injury epidemiology study (Owoeye et al., 2020), participants self-reported any knee or ankle injuries weekly, including patellar and Achilles tendinopathy on adapted Oslo Sports Trauma Research Centre Knee and Ankle Questionnaires. These questionnaires have been used in previous studies, and the adapted Oslo Sports Trauma Research Centre Knee Questionnaire is a valid tool for reporting patellar tendinopathy (Owoeye et al., 2018, 2019; Ghali et al., 2020). At study completion, the study physical therapist reviewed all injury data to remove duplicate injuries and classify injuries into either acute or overuse onset (Owoeye et al., 2020).

### Workload Data Processing

For each participant and each session, workload variables were calculated, including jump count (N) = number of jumps; jump height (centimeters) = the height of each jump summed across all jumps; weighted jump height (centimeters) = the height of each jump, raised to the power 9 based on the slope of the stress-life curve for the tendon (Edwards, 2018), then summed across all jumps and later raised to the power (1/9); session RPE (sRPE) in arbitrary units (AU) = RPE times the duration of the session in minutes. All data processing and analysis steps were conducted using built-in functions and custom MATLAB software (9.6.0.1335978 (R2019a) Update 8, Mathworks, Inc., Natick, MA, USA).

When workload data were missing for any reason (e.g., no data recorded that session, equipment malfunction, individual did not wear jump counter and/or report RPE, etc.), the missing workload variables for that session were imputed according to the following steps (Benson et al., 2021). For each workload variable, the workload for all non-missing participant-sessions was expressed relative to the duration of the session in hours. For each participant-session, eight features based on the context of the participant and session were computed: Value from the previous session; Mean of five previous sessions; Mean of all other sessions for the given individual; A random (*rand* function in MATLAB) value between 0 and the individual's maximum of all sessions, with equal probability of selecting any value within the range; A random value between 0 and the individual's maximum of all sessions, with the probability of selecting a value was based on the probability density function (*normpdf* function in MATLAB); Mean of all sessions for all participants on all teams of the same sex; Mean of all known values for the same team and the same session; Mean of all known values for the same team and the same session, weighted based on the mean ratios between the participants for known participant-sessions. The known values and features were used to train a least-squares boosted regression tree ensemble (*fitrensemble* function in MATLAB; number of learning cycles: 30, minimum leaf size: 8, learning rate: 0.1) to predict workload. The model was then used to impute all missing values of the given workload variable per hour and then multiplied by the missing session duration.

Each workload variable for each participant was summed across all sessions for each week (Monday–Sunday) of the season. Then, the previous 1-, 2-, 3-, and 4-week workloads were summed for each participant-week. The accumulated weighted jump height values were raised to the power (1/9) to match the units of cm for jump height (Edwards, 2018; Firminger et al., 2020). This resulted in a set of 16 accumulated workload variables for participant-weeks from the fifth week of the season until the end of the season.

### Analyses

Participant demographics were summarized using means and standard deviations for numerical data and frequencies and proportions for categorical data. For all injuries (i.e., upper and lower extremity; acute and overuse), the number of participants and injuries were reported.

The relationship between workload and injury was examined separately for three injury classifications: patellar or Achilles tendinopathy, overuse lower extremity injury, and any lower extremity injury. The injury classifications include the previous classification; overuse lower extremity injury includes all patellar or Achilles tendinopathy plus other overuse lower extremity injuries, and any lower extremity injury includes all overuse lower extremity plus acute lower extremity injuries. The week of first injury onset was identified for all participants with the specified injury, and the injury incidence rate (# injuries/100 participants/week) was plotted for each week of the season. For participants who did not experience the specified injury, the last week where they did not experience any injury was identified, which was the last week of the season if they were never injured. Recurrent injuries were not included in the analyses of the relationship between workload and injury.

To visualize trends in workload and injury over the season, the previous 1-, 2-, 3-, and 4-week mean accumulations of each workload variable were plotted as an ensemble average for all participants until the last week where they did not experience any injury. The same workload variables were plotted separately for individuals with patellar or Achilles tendinopathy, any other overuse lower extremity injury, and any acute lower extremity injury.

Each workload variable (jump count, jump height, weighted jump height, and sRPE) and accumulation (previous 1-, 2-, 3-, and 4-week workloads) were normalized across all participant-weeks to a mean of 0 and variance of 1. PCA (*pca* function in MATLAB) was used to explain the variance in the accumulated workload variables throughout the season. The first mode of variation, called a principal component (PC), explains the greatest amount of variance within the dataset. Subsequent PCs are identified in decreasing order of the variance explained for the dataset and are uncorrelated with the previous PCs, meaning each PC represents a unique aspect of the data. Often, a large percentage of the variance in the dataset can be explained by just a few PCs, and the smallest number of PCs that represented at least 90% of the variance in the dataset were retained (Jackson, 2003). The extent to which each variable is associated with the PCs is called the loading. The relationship between variables can be observed by how they load on the PCs, as similar variables will have similar loading. Thus, the mode of variation that a PC represents can be described by the variables that have the highest magnitude of loading (positive or negative) on that PC. The nature of the PCs was determined by plotting the loadings of each variable for each retained PC. Additionally, the original variables at each data point can be replaced by a smaller set of dimensionless PC scores, one for each PC. PC scores for each participant-week were computed, and the original workload variables representing the 5th and 95th percentile of the PC scores were plotted to illustrate the range of each retained PC (Brandon et al., 2013).

Using the uncorrelated retained PCs to represent workload, the difference in workload between injured and uninjured participants was examined for each injury classification. Using the PCs that represented the most variance in the dataset (PC1 and PC2), a scatterplot of the PC scores for every participant-week demonstrated how the workload of injured participants in the week before the injury was distributed relative to the workload for all participant-weeks of uninjured participants. The PC scores of injured participants for the week before injury were plotted with the mean of the PC scores for all weeks until the last week, where uninjured participants did not experience any injury, and differences were reported descriptively. Because the dataset contained multiple participant-weeks from each participant, a sensitivity analysis showed that the PCA results from the full dataset were similar to results from random subsets of the data that contained just one participant-week from each participant, so the full dataset was used.

## Results

### Participant Demographics and Injuries

Forty-nine [24 females, 25 males; age 16.5 (0.6) years; mass 66.6 (11.1) kg; height 1.74 (0.11) m; leg length 0.95 (0.07) m; injury in previous 12 months: 16 (32.7%) yes, 33 (67.3%) no] participants completed injury surveillance and workload monitoring throughout the season. Twenty-eight (57%) participants experienced a total of 44 injuries, with 37 (84%) injuries occurring in the lower extremity. There were seven overuse ankle injuries (two Achilles tendinopathies) and 14 overuse knee injuries (nine patellar tendinopathies) throughout the season (Table 1). For three reported injuries (two acute ankles and one overuse shin), the week of onset was not recorded. As such, these participants were excluded from the subsequent analyses.

**Table 1:** Total reported unique lower extremity injuries during the season.

**Injury type**	**Number of participants**	**Number of injuries**
Any lower extremity	26	37
Acute lower extremity	10	11
Overuse lower extremity	18	26
Overuse knee	14	14
Patellar tendinopathy	9	9
Overuse ankle	7	7
Achilles tendinopathy	2	2

**Figure 1:**
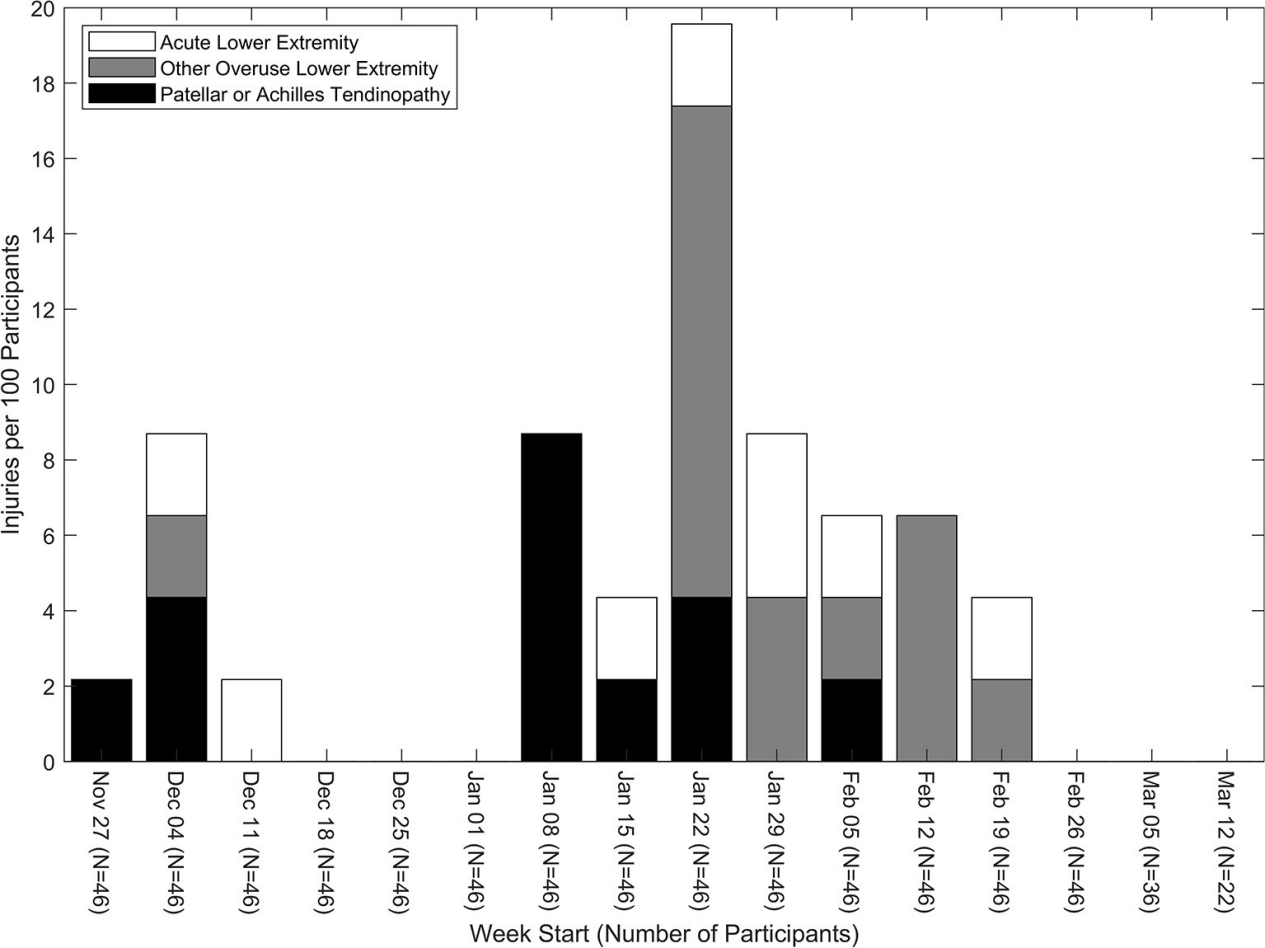
Number of injuries in each injury classification per 100 participants for each week of the season.

The injury incidence rate (# injuries/100 participants/week) in each week was highest in the week of January 22 (Figure 1). There were seven participants with patellar tendinopathy and no participants with Achilles tendinopathy as the first injury from the fifth week of the season until the end of the season. In the same timeframe, there were 13 participants with overuse lower extremity injuries (seven patellar tendinopathies and six other overuse lower extremity injuries) and 17 participants with any lower extremity injury (seven patellar tendinopathies, six other overuse lower extremity injuries, and four acute lower extremity injuries).

### Workload Patterns

**Figure 2:**
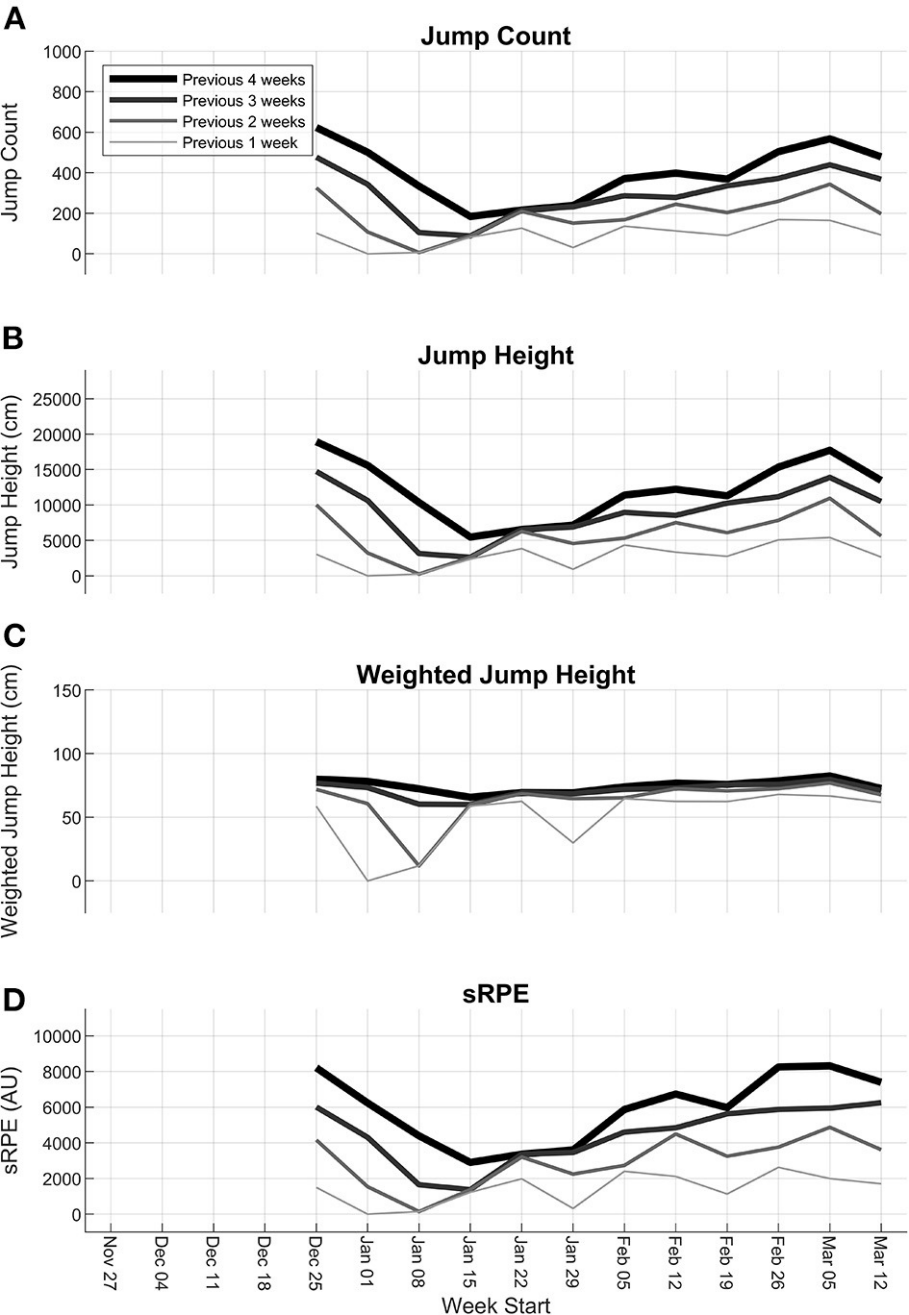
Mean 1-, 2-, 3-, and 4-week workloads of uninjured participants throughout the season for **(A)** jump count, **(B)** jump height, **(C)** weighted jump height, and **(D)** sRPE.

Jump data were imputed for 26–57% of participant-sessions, and RPE data were imputed for 34–62% of participant sessions across the four teams. There were 518 participant-weeks from the fifth week of the season until the end of the season. Plots of the workload variables over the season indicate decreases in workload that correspond with breaks in the schedule (Figure 2). The low values for the previous 1-week workload during the weeks of January 1, January 8, and January 29 represent breaks during the weeks of December 25 (holiday), January 1 (holiday), and January 22 (exams), respectively. The pattern of the previous 1-, 2-, 3-, and 4-week workloads of uninjured participants is similar throughout the season for jump count, jump height, and sRPE, with increasing workload magnitude as the number of accumulated weeks increases, and similar trends throughout the season. The weighted jump height plot reflects a consistent workload magnitude for each number of accumulated weeks and throughout the season, aside from the weeks that correspond with breaks in the schedule.

#### Jump Count and Jump Height

**Figure 3:**
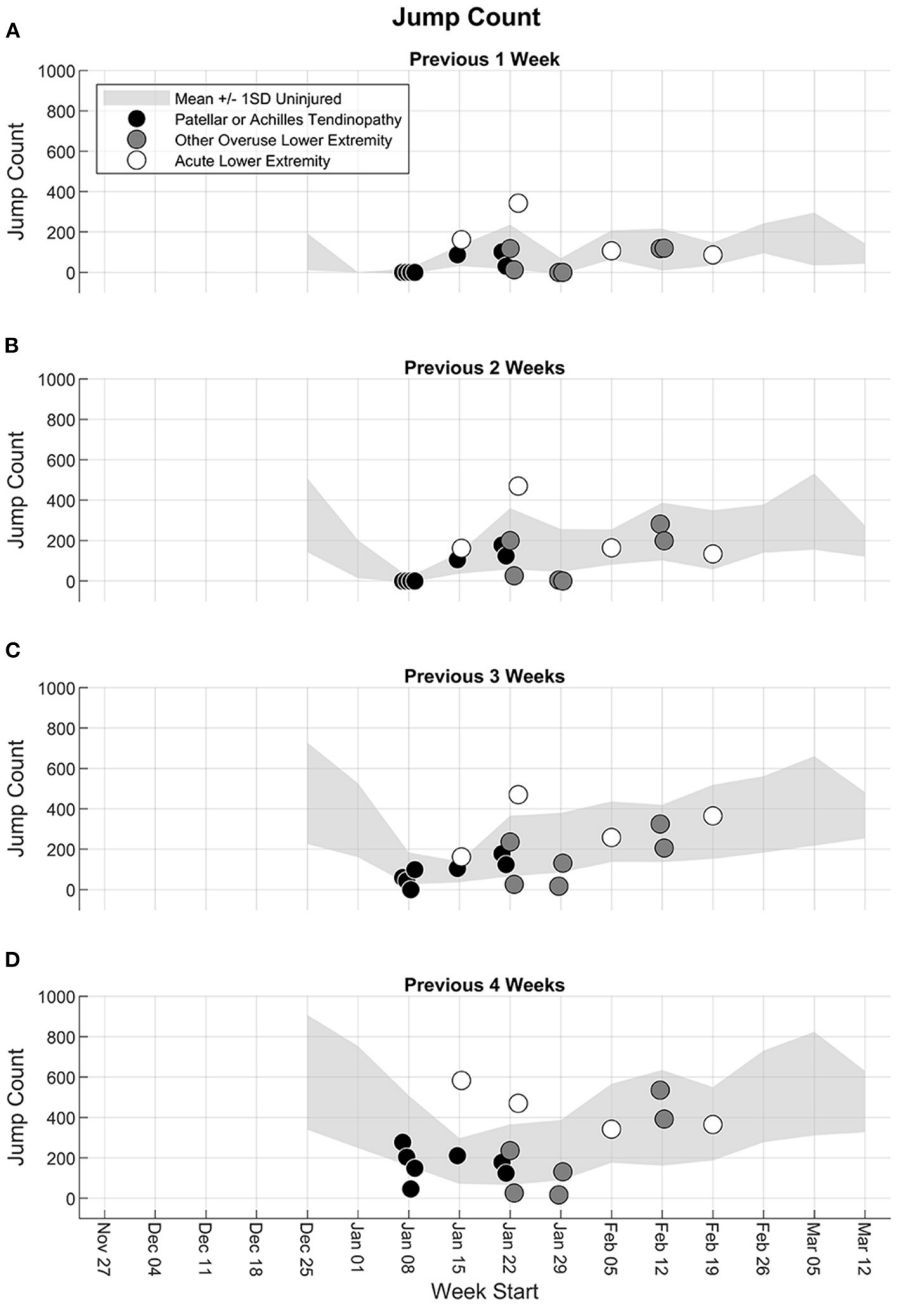
Accumulated jump count from the fifth week to the end of the season for the previous **(A)** 1 week, **(B)** 2 weeks, **(C)** 3 weeks, and **(D)** 4 weeks. Light gray shaded band represents the mean ± 1 standard deviation (SD) of all participants until the last week where they did not experience any injury. Participants with a lower extremity injury are shown separately in the week in which their first injury was reported, and the injury type is indicated as patellar or Achilles tendinopathy (black circle), other overuse lower extremity injury (dark gray circle), or acute lower extremity injury (white circle).

**Figure 4:**
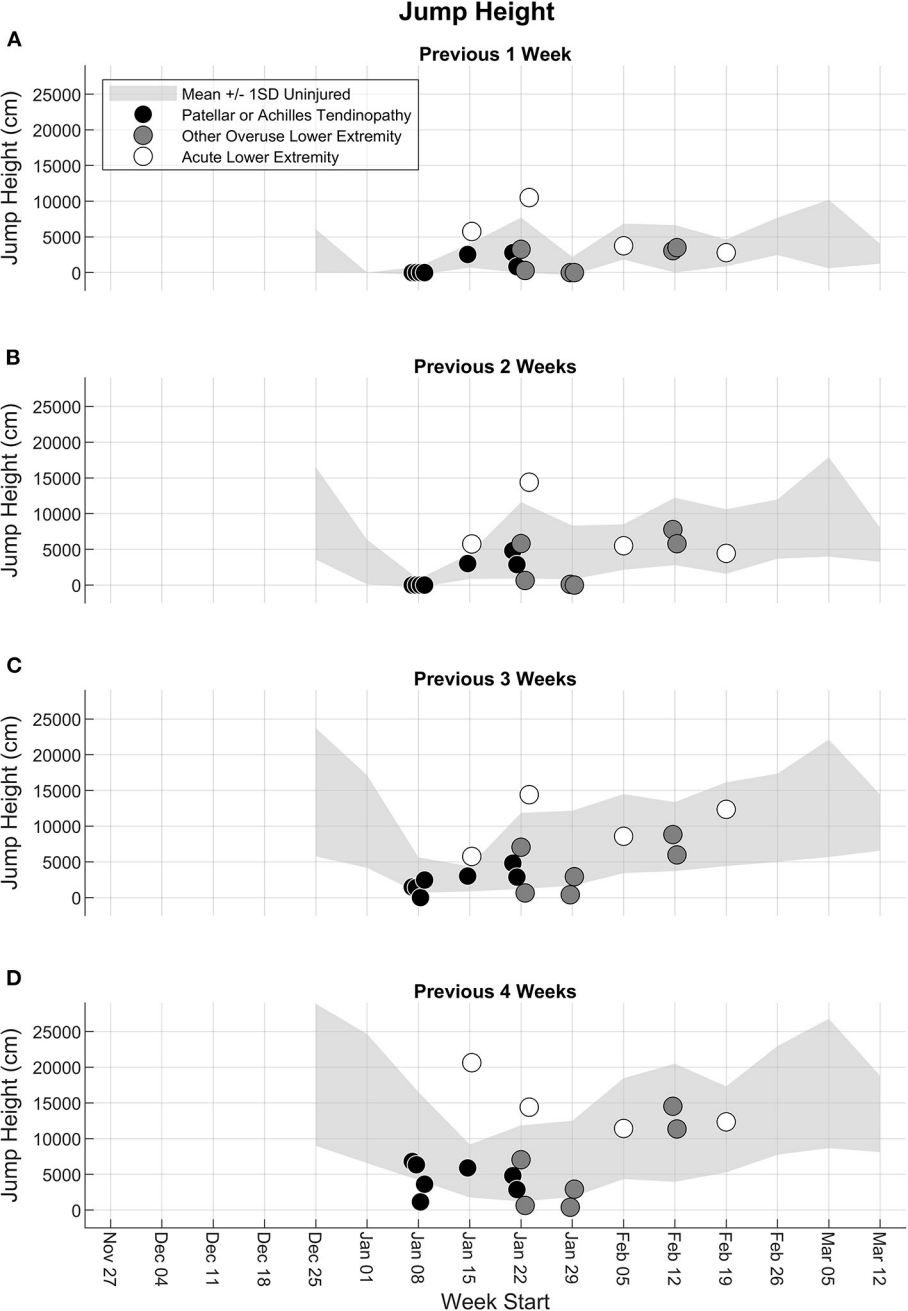
Accumulated jump height from the fifth week to the end of the season for the previous **(A)** 1 week, **(B)** 2 weeks, **(C)** 3 weeks, and **(D)** 4 weeks. Light gray shaded band represents the mean ± 1 standard deviation (SD) of all participants until the last week where they did not experience any injury. Participants with a lower extremity injury are shown separately in the week in which their first injury was reported, and the injury type is indicated as patellar or Achilles tendinopathy (black circle), other overuse lower extremity injury (dark gray circle), or acute lower extremity injury (white circle).

The previous 1-week jump count for participants who experienced a lower extremity injury was within ±1 standard deviation of the mean of the previous 1-week jump count of uninjured participants, except for two participants with acute lower extremity injury who had a greater previous 1-week jump count (Figure 3A). As the number of accumulated weeks increased from 2 to 4, the participants with patellar or Achilles tendinopathy or other overuse lower extremity injury around the time of the breaks in the schedule (weeks of January 8–29) had a jump count that was close to or lower than 1 standard deviation below the mean of the uninjured participants (Figures 3B–D). The two participants with acute lower extremity injury and a large previous 1-week jump count also had a large accumulated jump count over the previous 2, 3, and 4 weeks. Two participants with overuse lower extremity injury in the week of February 12 had previous 1-, 2-, 3-, and 4-weeks jump counts within ±1 standard deviation of the mean of uninjured participants. The jump height workload patterns for both the injured and uninjured participants were similar to the jump count workload patterns (Figure 4).

#### Weighted Jump Height

**Figure 5:**
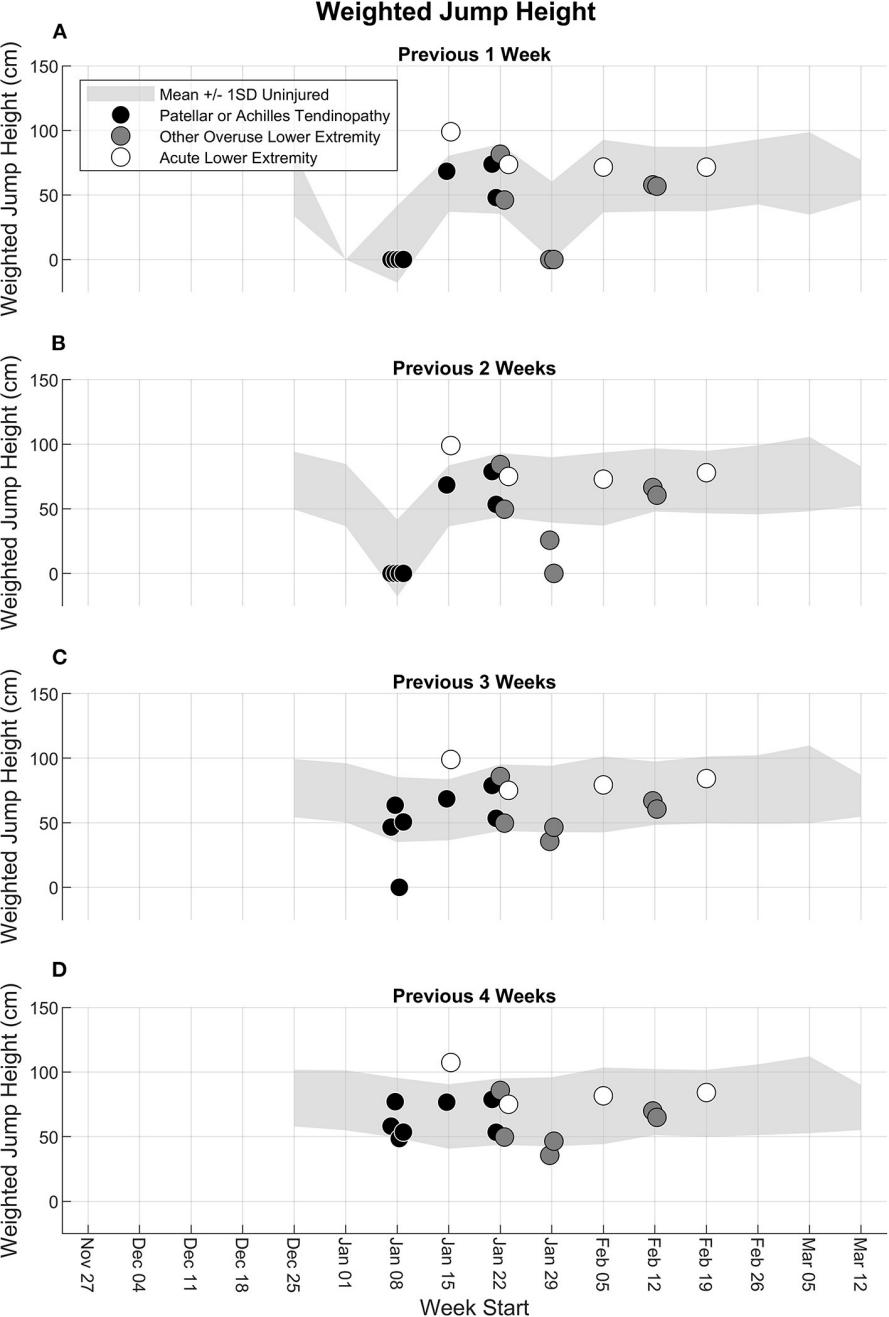
Accumulated weighted jump height from the fifth week to the end of the season for the previous **(A)** 1 week, **(B)** 2 weeks, **(C)** 3 weeks, and **(D)** 4 weeks. Light gray shaded band represents the mean ± 1 standard deviation (SD) of all participants until the last week where they did not experience any injury. Participants with a lower extremity injury are shown separately in the week in which their first injury was reported, and the injury type is indicated as patellar or Achilles tendinopathy (black circle), other overuse lower extremity injury (dark gray circle), or acute lower extremity injury (white circle).

The weighted jump height for participants who experienced a lower extremity injury was distributed within ±1 standard deviation of the mean of the uninjured participants for all weeks, with a few exceptions (Figure 5). Some, but not all, participants with patellar or Achilles tendinopathy or other overuse lower extremity injury that had a low 2-, 3-, and 4-week jump count and jump height relative to uninjured participants also had low 2-, 3-, and 4-week weighted jump heights. A participant with patellar or Achilles tendinopathy in the week of January 8 had a 3-week weighted jump height that was lower than 1 standard deviation below the mean of the uninjured participants but was at the low end of the range for the previous 1, 2, and 4 weeks. Likewise, two participants with overuse lower extremity injury in the week of January 29 had a 2-week weighted jump height that was lower than 1 standard deviation below the mean of the uninjured participants but were at the low end of the range for the previous 1, 3, and 4 weeks.

#### Session Rating of Perceived Exertion

**Figure 6:**
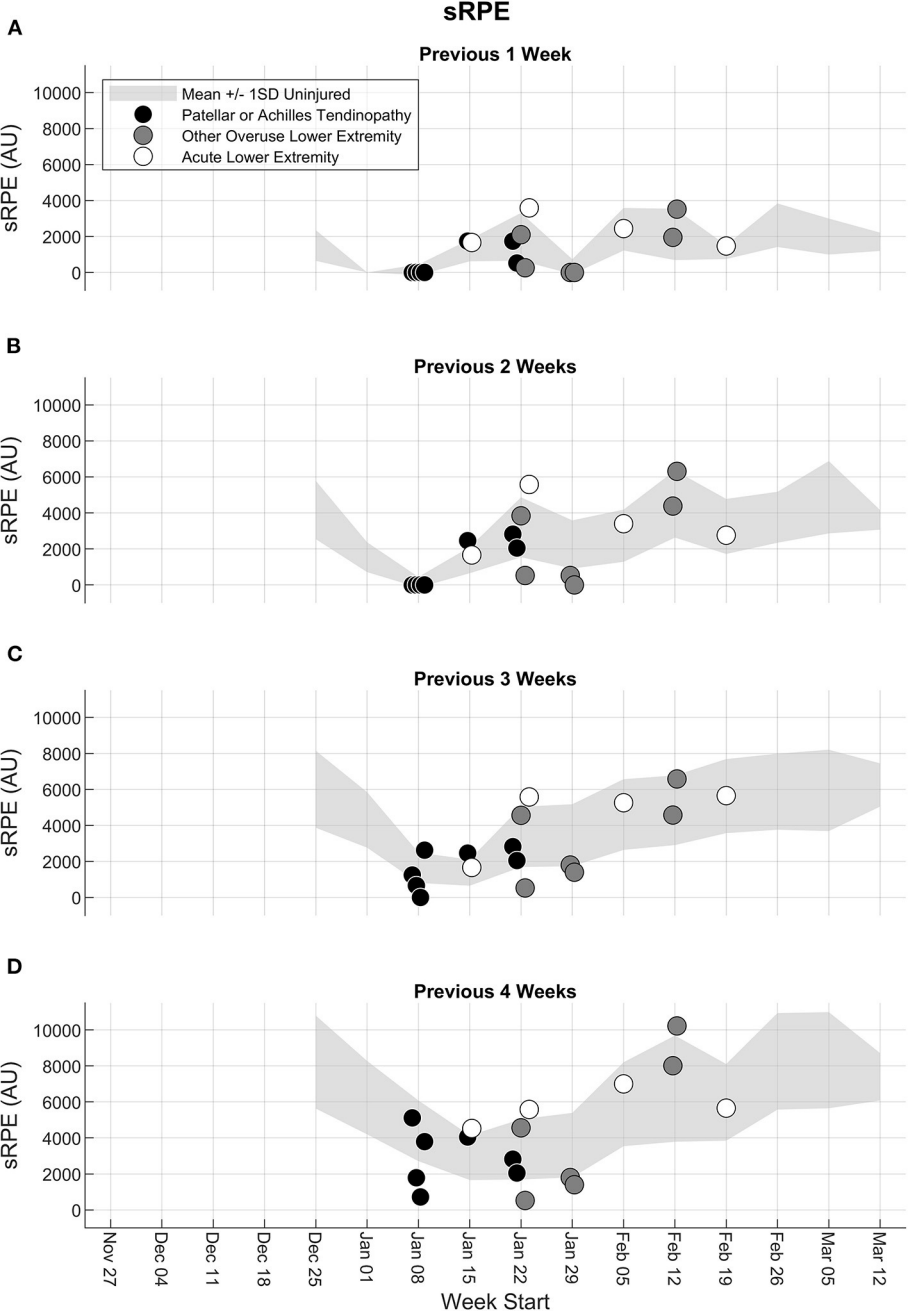
Accumulated sRPE from the fifth week to the end of the season for the previous **(A)** 1 week, **(B)** 2 weeks, **(C)** 3 weeks, and **(D)** 4 weeks. Light gray shaded band represents the mean ± 1 standard deviation (SD) of all participants until the last week where they did not experience any injury. Participants with a lower extremity injury are shown separately in the week in which their first injury was reported, and the injury type is indicated as patellar or Achilles tendinopathy (black circle), other overuse lower extremity injury (dark gray circle), or acute lower extremity injury (white circle).

The sRPE workload patterns were similar to the jump count and jump height patterns in that some, but not all, participants with patellar or Achilles tendinopathy or other overuse lower extremity injury who had a low 2-, 3-, and 4-week jump count and jump height relative to uninjured participants also had a low 2-, 3-, and 4-week sRPE (Figure 6). However, some injured participants with a low jump count and jump height had an sRPE close to 1 standard deviation above the mean of the uninjured participants for the previous 1, 2, 3, and 4 weeks.

### Workload Modes of Variation

**Figure 7:**
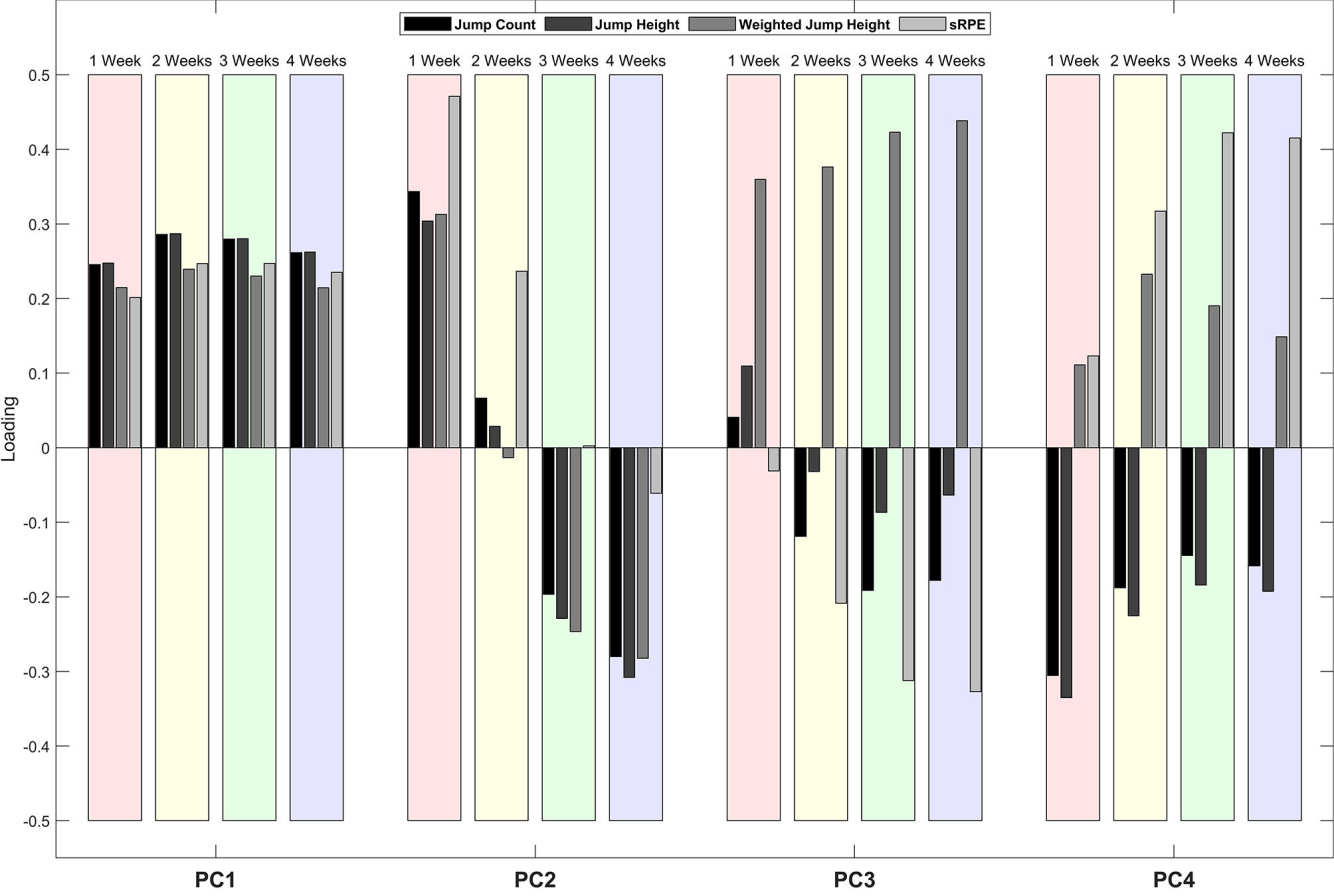
Loading of each workload variable (jump count, jump height, weighted jump height, and sRPE) and accumulation (previous 1-, 2-, 3-, and 4-week workloads) on each of the retained PCs. A positive value for loading indicates a positive association between the workload variable and the PC. Variables with a higher loading magnitude indicate a stronger association with the PC.

Four retained PCs represented 92.1% of the variance in the 16 workload variables across all participant-weeks (Table 2). The loading of the workload variables on each retained PC indicated that PC1 represents the overall workload magnitude and the other retained PCs represent differences between workload variables and the number of weeks over which they are accumulated (Figure 7).

**Table 2:** The retained PCs and their percent of total variance explained.

**PC**	**Variance explained (%)**	**Description**
PC1	64.8	High workload magnitude for all variables and all weeks
PC2	12.3	High 1-week workload for all variables;
		Low 3- and 4-week jump count, jump height, and weighted jump height
PC3	8.6	High weighted jump height for all weeks;
		Low 2-, 3-, and 4-week jump count, jump height, and sRPE
PC4	6.4	High weighted jump height and sRPE for all weeks;
		Low jump count and jump height for all weeks

*The description is based on the loading of the workload variables on each PC*.

**Figure 8:**
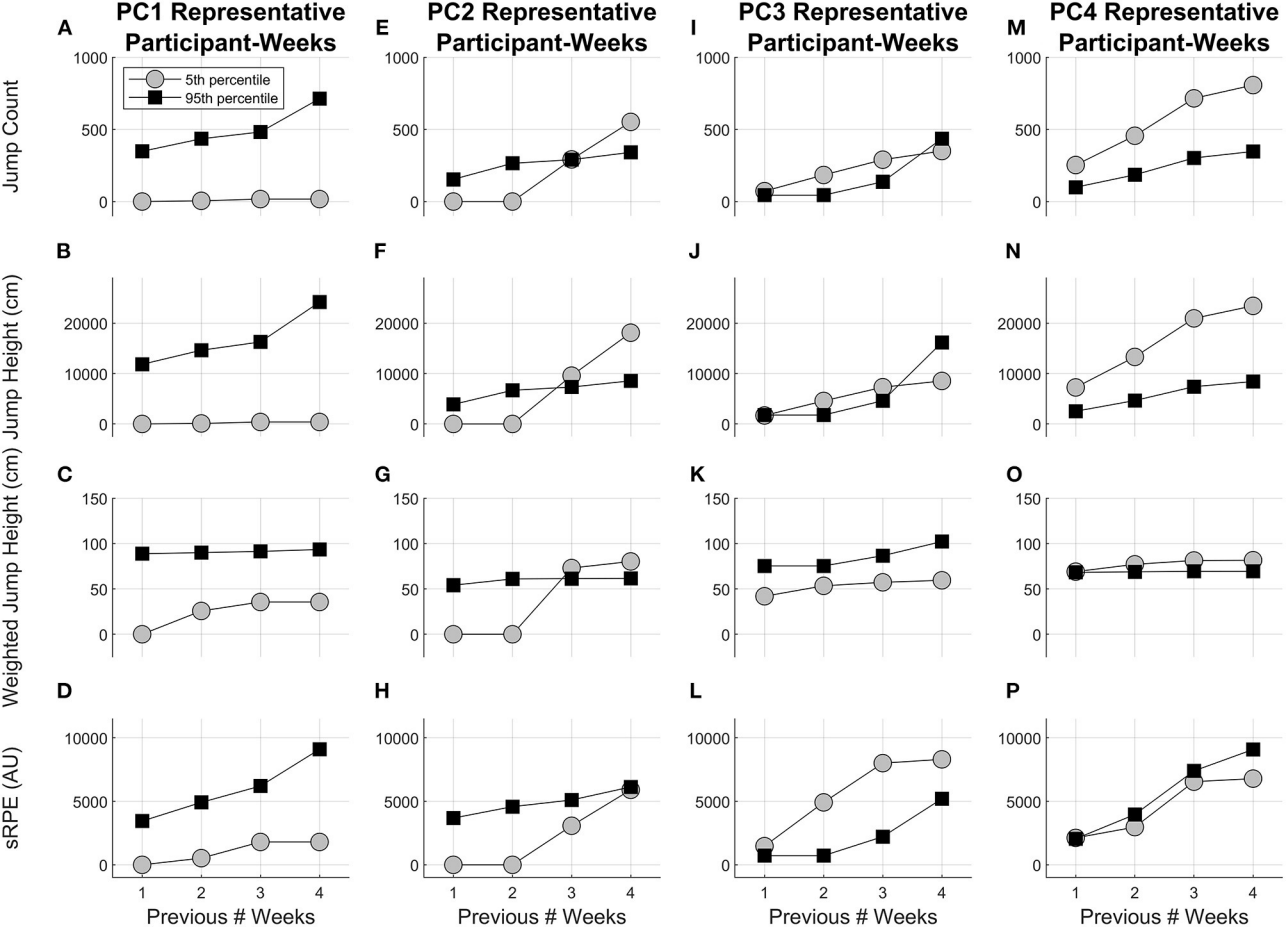
Original workload variables for the 5th and 95th percentile representative participant-weeks for **(A–D)** PC1, **(E–H)** PC2, **(I–L)** PC3, and **(M–P)** PC4. Fifth percentile represents a low PC score, and 95th percentile represents a high PC score for a given PC. Note that the representative participant-weeks are different for each PC.

PC1 describes a high workload magnitude for all variables and all weeks. This is demonstrated in Figures 8A–D, where the participant-week that represents the 5th percentile of the PC1 score has lower workload values than the participant-week that represents the 95th percentile of the PC1 score for all variables and all weeks. PC2 describes a high previous 1-week workload for all variables along with low previous 3- and 4-week jump counts, jump heights, and weighted jump heights. The participant-weeks that represent the 5th and 95th percentiles of the PC2 score cross at the previous 3-week accumulation for jump count, jump height, and weighted jump height, and a similar trend is shown for sRPE (Figures 8E–H). PC3 describes a high weighted jump height for all weeks and low previous 2-, 3-, and 4-week jump counts, jump heights, and sRPEs. The participant-week that represents the 5th percentile of the PC3 score has a lower weighted jump height than the participant-week that represents the 95th percentile of the PC3 score for all weeks and workload values that are greater than or similar to the participant-week that represents the 95th percentile of the PC3 score for jump count, jump height and sRPE (Figures 8I–L). PC4 describes a high weighted jump height and sRPE and a low jump count and jump height for all weeks. The participant-week that represents the 5th percentile of the PC4 score has similar weighted jump height and sRPE as the participant-week that represents the 95th percentile of the PC4 score for all weeks and workload values that are greater than the participant-week that represents the 95th percentile of the PC4 score for jump count and jump height (Figures 8M–P).

### Workload and Injury

**Figure 9:**
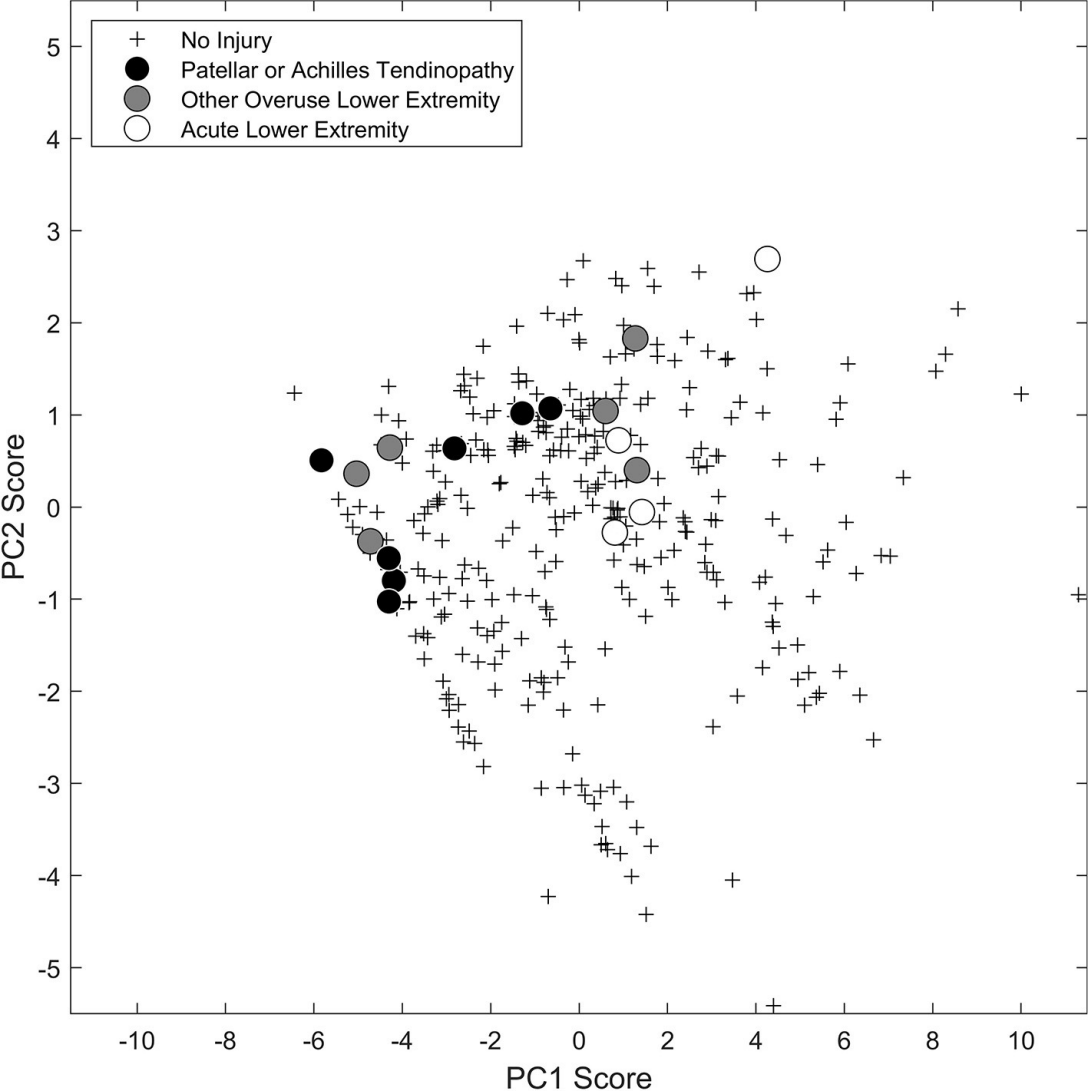
Scatterplot of the PC1 and PC2 scores for all participant-weeks with no injury (+) and the week before injury for patellar or Achilles tendinopathy (black circle), other overuse lower extremity injury (dark gray circle), or acute lower extremity injury (white circle).

From the scatterplot of the PC1 and PC2 scores for every participant-week, those representing injured participants in the week before injury tended to cluster in the upper left quadrant indicating a low PC1 score and a high PC2 score (Figure 9). Specifically, all participants with patellar or Achilles tendinopathy and half of the other overuse lower extremity injuries had a negative PC1 score, representing a low workload magnitude for all variables in the previous 1, 2, 3, and 4 weeks. Additionally, four of the participants with patellar or Achilles tendinopathy and five of the other overuse lower extremity injuries had a positive PC2 score, representing a high previous 1-week workload for all variables along with low previous 3- and 4-week jump counts, jump heights, and weighted jump heights. The remaining participants with patellar or Achilles tendinopathy or other overuse lower extremity injury that did not have a negative PC1 score and a positive PC2 score had scores close to zero. Likewise, three participants with acute lower extremity injury also had PC1 and PC2 scores close to zero.

**Figure 10:**
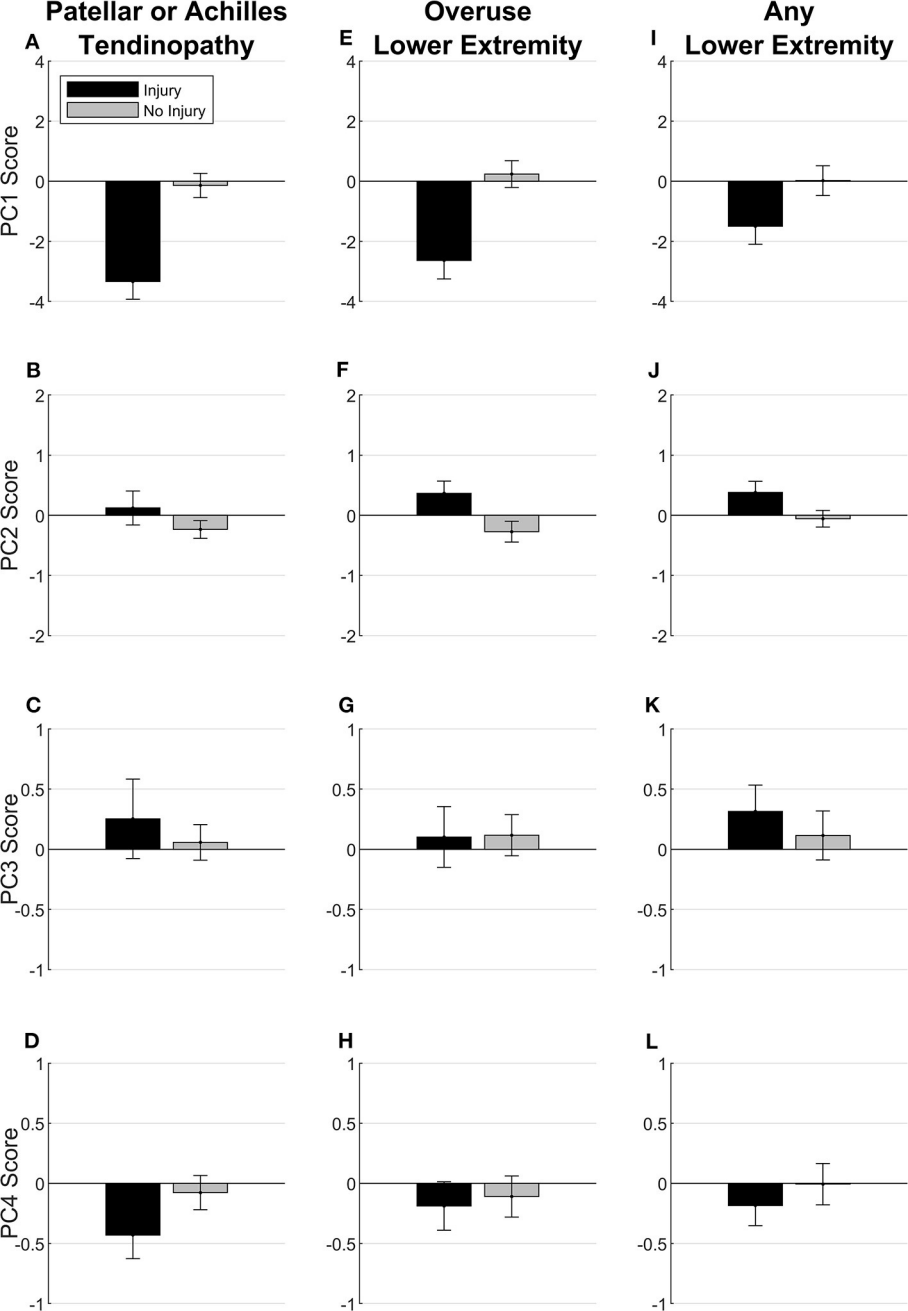
Mean and standard error of PC scores for injured participants representing workload in the week before injury and the mean of all weeks until the last week where they did not experience any injury for uninjured participants. Visual comparisons are made for three separate injury classifications: **(A–D)** patellar or Achilles tendinopathy, (**E–H**) overuse lower extremity injury, (**I–L**) any lower extremity injury.

PC1 score was lower, representing a low workload magnitude for all variables in the 1, 2, 3, and 4 weeks before injury compared with the weeks before no injury for patellar or Achilles tendinopathy and overuse lower extremity injuries and, to a lesser extent, any lower extremity injury (Figure 10). PC2 score was higher, representing a high previous 1-week workload for all variables along with low previous 3- and 4-week jump counts, jump heights, and weighted jump heights before injury compared with the weeks before no injury for overuse lower extremity injuries and any lower extremity injury but not for patellar or Achilles tendinopathy. There were no observed differences between injured and uninjured participants for PC3 (high weighted jump heights for all weeks and low previous 2-, 3-, and 4-week jump counts, jump heights, and sRPEs) or PC4 (high weighted jump heights and sRPEs and low jump counts and jump heights for all weeks).

## Discussion

In this study, we identified four distinct modes of variation in workload metrics accumulated over 1, 2, 3, and 4 weeks throughout a youth basketball season and observed differences among the modes of variation in workload metrics between participants before the injury and uninjured participants. One of the modes of variation indicated that weighted jump height is a unique metric relative to unweighted measures of external load and internal load; however, injured and uninjured participants were similar on the mode of variation in which the weighted jump height variables had the highest loading. The differences related to injury suggested that participants with patellar or Achilles tendinopathy and overuse lower extremity injuries had a low workload magnitude for all variables, including weighted jump height, in the 1, 2, 3, and 4 weeks before the injury. Additionally, a time difference was identified where participants with overuse and any lower extremity injury had a high previous 1-week workload for all variables relative to low previous 3- and 4-week jump counts, jump heights, and weighted jump heights.

### Weighted Jump Height

The weighted jump height metric was designed as a surrogate measure of tendon damage such that higher magnitude jumps had a greater contribution to workload than the frequency of jumps. Thus, the accumulation of weighted jump height followed a different pattern throughout the season than the other measures of external load. Over a given number of weeks, the accumulated weighted jump height was dominated by the highest jumps, which led to only small differences in accumulated workload as the number of weeks increased. In contrast, the accumulated jump count, jump height, and sRPE metrics increased as the number of weeks increased. PC3 identified weighted jump height as a unique workload metric, as a high PC3 score corresponded with a high weighted jump height even as the other workload metrics were low. PC4 also demonstrated how weighted jump height and sRPE could be independent of the unweighted external load metrics. Because the greatest magnitude jumps dominate the weighted jump height, the lack of difference between injured and uninjured participants for PC3 and PC4 suggests that before the injury, athletes had a similar jump height for their highest jumps as uninjured participants. Although the PCs that represented a high weighted jump height and low values of unweighted external load were not different between injured and uninjured participants, it is possible that the comparison with only seven patellar tendinopathies and six other overuse lower extremity injuries may not have been enough cases to identify differences between injured and uninjured athletes. Furthermore, the youth basketball players were not always executing maximal jumps during their practices and games; thus, the weighted jump height metric should be investigated in sports with a greater proportion of maximal jumps, such as volleyball. Nevertheless, the weighted jump height metrics had similar loading to other measures of external load in PC1 and PC2, which did show differences between injured and uninjured participants, suggesting that jump magnitude may be used to monitor injury risk. Additionally, an injury may influence the ability to generate a high magnitude jump, in which case weighted jump height could be used to monitor jumping ability during rehabilitation following injury.

### Workload and Injury

The observed differences between injured and uninjured athletes for PC1 suggest that all workload metrics accumulated over 1, 2, 3, and 4 weeks were lower before overuse of lower extremity injury, including patellar or Achilles tendinopathy, than for uninjured athletes. The differences for PC2 indicate that the injured athletes had a lower workload accumulated over 3 and 4 weeks but a higher 1-week workload. Without calculating an ACWR, the differences between injured and uninjured athletes for PC2 indicate that a low workload accumulation over 3 and 4 weeks coupled with a high 1-week workload could contribute to injury risk. Both results highlight the importance of workload accumulated over several weeks as protection against injury. The overall low levels of accumulated workload may indicate underlying pain not reported as an injury while an overuse injury is developing. It also seems that the low accumulated workload in this study may be a byproduct of scheduling and training decisions, as the lowest 1-week workloads averaged across all participants corresponded with school breaks and remained relevant as the cumulative workload was summed over the subsequent weeks. Therefore, youth sport coaches and athletes may consider maintaining workload levels during breaks in the schedule to avoid dramatic decreases in accumulated workload.

These findings are consistent with previous studies that identified an increased risk of injury was associated with both low chronic workloads and high acute relative to chronic workloads (Hulin et al., 2016a,b; Colby et al., 2017; Stares et al., 2018; Bowen et al., 2020). Several review articles have summarized other studies that also demonstrated an association between high ACWR and injury (Drew and Finch, 2016; Jones et al., 2017; Eckard et al., 2018; Benson et al., 2020a; Griffin et al., 2020). It should be noted that in most cases, the original studies on the workload–injury relationship investigated many combinations of workload metrics and methods for accumulating workload, resulting in the identification of multiple significant associations (Benson et al., 2020a). In contrast to the results of this study, significant associations between workload and injury have also been reported when the chronic workload is high (Colby et al., 2014, 2018; Bowen et al., 2017; Malone et al., 2017; Esmaeili et al., 2018; Jaspers et al., 2018), and embedded in the studies that investigated many combinations of workload and accumulation methods are reports of no association between ACWR and injury (Impellizzeri et al., 2020c). Various inconsistencies across studies, including different sports, levels of competition, injury definitions, and types of workload, in addition to selective reporting bias, contribute to the lack of consensus on the workload–injury relationship. The PCA approach used in this study for identifying distinct modes of variation within a workload may be helpful for understanding workload differences between injured and uninjured athletes.

### Limitations

A limitation of this study was the lack of subject-specific adjustments to the use of jump height for representing jump magnitude. Absolute jump height (centimeters) was used for the jump height metric and weighted jump height calculation. It is possible that normalizing jump height based on a participant's jumping ability (e.g., maximum jump height) may more accurately demonstrate how jump magnitude influences injury risk. Although the weighted jump height metric was designed to reflect the relative importance of loading magnitude overloading cycles in the accumulation of tissue damage, the same calculation (jump height raised to the power 9) was applied universally to all participants. Models of overuse injury can be improved with subject-specific parameters (Edwards, 2018). For example, tendon properties change based on age (Heinemeier et al., 2013), suggesting that the appropriate value for weighting jump height may differ between the youth in this study and other populations. Furthermore, it is possible that time intervals other than 1, 2, 3, and 4 weeks would better represent the accumulation of workload. Although our findings are consistent with and expand on previous studies examining acute loads at 1 week and chronic loads at 4 weeks before the injury, future longitudinal studies may consider if timeframes less than 1 week and/or greater than 4 weeks are also relevant.

By avoiding the calculation of an ACWR, we avoided the mathematical shortcomings associated with ACWR analyses. However, as with other attempts to evaluate the effects of accumulated workload, the initial weeks of the season could not be included in the analysis. In this case, the workload patterns associated with six injuries (three patellar or Achilles tendinopathy, one other overuse lower extremity injury, and two acute lower extremity injuries) could not be examined, as the injuries occurred within the first 3 weeks of the season. Overall, the small number of reported injuries is also a limitation, and a larger-scale study should be conducted to confirm these findings.

In this study, PCA eliminated the redundancy created by accumulating similar workload variables over several iterations of multiple weeks. The result was modes of variation that described overall workload magnitude, differences between weeks of accumulated workload, and unique aspects of workload metrics, including differences between weighted and unweighted and external and internal measures of a load. A limitation to this approach, however, is that PCA of other workload datasets may not identify the same modes of variation. The workload dataset used for the PCA in this study contained multiple weeks of data from the same participants, a large portion of which was imputed, and the within- and between-player variability from this dataset may differ in future analyses of player workload. Additionally, the magnitude of the original workload variables will be different for other athletes, sports, and levels of competition. In this study, the PCs represented the variance in this dataset, and individual participant-weeks scored higher or lower on each PC relative to the other participant-weeks in the dataset. Furthermore, an assumption of PCA is the independence of observations, which was not the case in this dataset that included participant-weeks from the same participants throughout the season and participants on the same team. Our analysis of multiple datasets, each with just one participant-week observation from each participant, revealed similar PCA results to the analysis of the full dataset. Nevertheless, methods to account for lack of independence in PCA could be explored. Future studies should consider the appropriate individual or composite metrics representing key modes of workload variation while avoiding redundancy in metrics or how they are accumulated.

## Conclusions

In conclusion, this study established weighted jump height as a workload metric that represents the cumulative damage experienced by tissues in the body that are subjected to repetitive loads. Without choosing *a priori* the workload metrics and methods for representing the accumulation of workload, it was shown that injured youth basketball athletes had a low workload accumulation over 3 and 4 weeks coupled with a high 1-week workload.

## Data Availability Statement

The aggregated data supporting the conclusions of this article will be made available by the authors, without undue reservation.

## Ethics Statement

The studies involving human participants were reviewed and approved by University of Calgary. Written informed consent to participate in this study was provided by the participants' legal guardian/next of kin.

## Author Contributions

OO, CS, and CE contributed to the study design. LB, OO, AR, and CS contributed to data collection, entry, and/or data cleaning. LB, OO, AR, and WE contributed to the data analysis and interpretation of study results. CE was the nominated PI for the larger cohort. All authors critically reviewed and edited the manuscript before submission.

## Conflict of Interest

The authors declare that the research was conducted in the absence of any commercial or financial relationships that could be construed as a potential conflict of interest.
